# Beneficial Effects of Glucagon-Like Peptide-1 Receptor Agonists in Patients With Asthma: A Literature Review

**DOI:** 10.7759/cureus.30812

**Published:** 2022-10-28

**Authors:** Meeta K Kanwar, Ramya Sunku, Faisal Alruwaili, Mariam A Mufti, Mukaila Raji

**Affiliations:** 1 Department of Internal Medicine, University of Texas Medical Branch, Galveston, USA; 2 Department of Internal Medicine-Division of Geriatrics & Palliative Medicine, University of Texas Medical Branch, Galveston, USA; 3 Department of Preventive Medicine and Population Health, University of Texas Medical Branch, Galveston, USA

**Keywords:** allergy, inflammation, obesity, diabetes, asthma, glucagon-like peptide-1 receptor agonist (glp-1 ra)

## Abstract

Glucagon-like peptide-1 receptor agonists (GLP-1 RAs) are approved to treat type 2 diabetes mellitus. The anti-inflammatory and anti-obesity properties of GLP-1 RAs as well as their moderating effects on multiple pathobiological pathways in asthma pathogenesis may decrease asthma exacerbations, improve quality of life, and decrease premature death among patients with asthma and co-morbid diabetes or obesity.

The aim of this literature review is to discuss evidence from basic science, human studies, and clinical trials to support the preferential use of GLP-1 RAs in asthma patients with co-occurring diabetes and obesity.

The preliminary data on the effect of GLP-1 RAs on asthma in patients with diabetes are promising and merit further trials and research studies.

## Introduction and background

What are GLP-1 (glucagon-like peptide-1) receptor agonists?

GLP-1 (glucagon-like peptide-1) is a neuroendocrine peptide that is secreted by intestinal L cells [1]. GLP-1 Receptor Agonists (GLP-1 RAs) like liraglutide, semaglutide, etc. are used to treat diabetes and euglycemic obesity. GLP-1 receptors (GLP-1R) are G-coupled protein receptors. Within the pancreas, they activate B cells via a cAMP mechanism to increase insulin secretion. GLP-1Rs are also found in the pulmonary vasculature, smooth muscle cells, and in type 2 alveolar cells [2]. Inhaled formulations of GLP-1 RAs were the first to be developed, and now we have injectable and oral formulations as well [3]. The most common use of GLP-1 RAs is in the treatment of diabetes, however, there are other potential benefits.

Anti-inflammatory pathways of GLP-1 receptor agonists

GLP-1 RAs have shown anti-inflammatory effects on pancreatic and adipose tissue which aid in reducing glucose levels in diabetics. Inflammation can be the cause of insulin resistance and beta-cell damage. Inflammation, therefore, plays a vital role in diabetes pathogenesis.

Specifically, GLP-1 RAs have been shown to reduce inflammation in multiple ways. Exendin-4, a GLP-1 RA, suppresses the expression of inflammatory genes such as NFkb1, NFkB2, and TNF receptors. Additionally, serine proteinase inhibitor-9 plays a role in the survival of cells against the attack from NK cells and cytotoxic T cells. These cells are directly involved in the destruction of beta-cells in type I diabetes. Exenatide also induces the expression of serine proteinase inhibitor-9 in islet cells. Therefore, GLP-1 RAs downregulate inflammatory mediators and the autoimmune response that causes the destruction of pancreatic islets cells. The use of GLP-1 RAs reduces the macrophages that are recruited to the adipose tissue and reduces the production of TNF-alpha, MCP-1, and IL-6 in the surrounding adipose tissue [4]. This is done via the inhibition of NF-Kb activation and phosphorylation of ERK1/2 and c-Jun N-terminal kinases. These mechanisms, in turn, increase insulin sensitivity [4].

To summarize, GLP-1 RAs can reduce inflammation by decreasing the production of proinflammatory cytokines, preserving pancreatic islet cells, and improving insulin sensitivity.

Diabetes and asthma co-occurrence

Individuals with diabetes, obesity, and metabolic syndrome have a different phenotype of asthma than those who have childhood asthma. There is a neutrophilic predominance in this case. In a small subsection of this population having neutrophilic-predominant asthma, some will be more resistant to steroids [5]. Alternative therapies used in the treatment of severe asthma include immunoglobins which are expensive. Common targets for these immunoglobins are IL-13 and TSLP (thymic stromal lymphopoietin). The risk of developing asthma increases with higher BMI, and those who are obese tend to have poorer outcomes. Insulin resistance potentiates the association between obesity and asthma [6]. Inflammation in obesity and asthma is a common pathway that could be targeted to reduce symptoms of both diseases. GLP-1 RAs are a nonsteroid alternative that could be used to treat diabetes and asthma [7]. Some of the common inflammatory mediators are the NLRP3 pathway and IL-6-mediated inflammation that affect both diseases [8].

GLP-1 receptor agonists and BMI reduction

Effects of GLP-1 RAs in weight reduction have been well documented, with several GLP-1 RAs (semaglutide and exenatide weekly injections) approved by FDA (Food and Drug Administration) for obesity management. Preliminary evidence suggests that GLP-1 RAs-associated weight loss improves patient-reported asthma outcomes. Interestingly, a small prospective cohort study showed that liraglutide therapy in asthmatic, obese, type 2 diabetic subjects led to fewer asthma exacerbations irrespective of significant weight loss (the cutoff in the study was 2.5kg weight loss in 52 weeks) [9].

GLP-1 receptor agonists and lungs

In rat model studies, GLP-1 receptors are present in high numbers in the lungs especially in the smooth muscle, submucosal glands of the trachea, and type 2 pneumocytes that produce surfactant. Due to the location of these receptors, GLP-1 RAs can have anti-inflammatory and anti-fibrotic effects on the lungs. Two studies showed an increase in surfactant production, an important process for lung development and prevention of lung collapse [10,11]. Additionally, the anti-inflammatory marker periostin, a marker of airway inflammation seen in asthmatics, is often decreased because of GLP-1 RA therapy [12]. This suggests that periostin levels can potentially be used to measure the anti-inflammatory effects of GLP-1 RAs, however, this claim needs further studies. The anti-fibrotic effects of GLP-1 RAs were shown in mice. Lung samples from the mice were injected with lipopolysaccharide. The same samples were then injected with the GLP-1 RA, vildagliptin. The results of flow cytometry in 28 days after injection showed an increase in the number of pulmonary vascular endothelial cells expressing alpha-smooth muscle actin or S100 calcium-binding protein A4 [13]. Additionally, endothelial-mesenchymal transition was downregulated due to vildagliptin, thus reducing fibrosis [13]. In summary, there are various mechanisms in which inflammation and fibrosis are reduced, specifically in the lungs of subjects on GLP-1 RAs with asthma.

## Review

What have human studies shown?

A retrospective cohort study found that patients on GLP-1 RAs compared to other medications to treat type 2 diabetes mellitus had a lower rate of asthma exacerbations [14,15]. However, the sample size was small. In another meta-analysis GLP-1 RAs were not found to influence asthma exacerbations although the sample size was small, and the database likely had underreported asthma exacerbations [2]. In conclusion, more human studies are needed.

What have animal studies shown?

In animal models, GLP-1 RAs have been shown to reduce airway inflammation, obstruction, and fibrosis. Specifically, the GLP-1 receptor plays a role in the inhibition of allergen-induced type 2 inflammation [16]. This occurs via the reduction of cytokine release, suggesting that GLP-1 receptor signaling may inhibit the allergic response in the lung.

Specifically, liraglutide reduces eosinophilic and IL-33-mediated airway allergic inflammation which is involved in Th2 immune response after an allergen challenge. Additionally, GLP-1 RAs relieved asthmatic airway inflammation via suppression of NLRP3 inflammasome activation in obese mice models [17].

In another study, liraglutide was used in animal models to test whether it improves fibrosis induced by bleomycin. Liraglutide decreases mRNA expression of collagen and restored ACE2 mRNA levels modulating activities of renin-angiotensin system (RAS) components to ultimately increase the production of surfactant proteins [18].

Exendin-4, specifically, has been shown to contribute to lung healing against oxidative stress caused by hyperglycemia. It also causes a reduction in fibronectin, preventing excessive collagen buildup in the lungs of diabetic mice [4].

Overall, GLP-1 RAs have been shown to be anti-inflammatory and potentially disease-modifying for asthmatics with diabetes in animal models (Tables 1, 2) [7]. GLP-1 agonists were also found to decrease eosinophilic-mediated secretion of IL4, IL8, and IL13 in mice with asthma [19].

**Table 1:** Anti-Inflammatory and Disease-Modifying Effects of GLP-1 RAs

Types of Effects of GLP-1 RAs in the Lung	
Anti-Inflammatory Effects:	Reduce eosinophilic and IL-33-mediated inflammation
	Suppress NLRP3 inflammasomes
	Decrease eosinophilic-mediated secretion of IL4, IL8, and IL13
Anti-Fibrotic Effects:	Restore ACE2 mRNA levels which modulate activities of RAS
	Reduce fibronectin thereby preventing excessive collagen buildup
	Decrease mRNA expression of collagen components to increase surfactant proteins
Anti-Oxidative Effects:	Contribute to lung healing against oxidative stress caused by hyperglycemia

**Table 2:** GLP-1 RAs Effects on Inflammation and Asthma

Paper/Reference	Study	Main Results
Voss et al. [1]	Animal model	GLP 1 and 2 have neuroprotective effects and can potentially be used for a wide variety of autoimmune diseases.
Wang et al. [2]	Meta-analysis	GLP1 RA did not affect the incidence of asthma.
Hohenegger [3]	Review Article	GLP1 Receptor agonists can be given in inhaled versions.
Oztay et al. [4]	Animal Model	Exendin-4, a GLP1 receptor agonists, was found to improve pulmonary edema, oxidative stress and lung injury in diabetic mice. Exendin-4 decreased hyperglycemic lung damage by reducing glucose-mediated oxidative stress. It was also found to increase lung injury by decreasing insulin signaling in the lungs.
McCravy et al. [5]	Animal Model	Patients with metabolic syndrome have been shown to have dysregulation of GLP1 and NO. Patients with asthma and metabolic syndrome may have a non-eosinophilic phenotype of asthma. Novel therapies are needed to treat this asthma phenotype.
Cardet et al. [6]	Review article	Relationship between Type 2 Diabetes and Asthma with insulin resistance.
Wu et al. [7]	Review article	GLP1 R Agonists have anti-inflammatory properties. GLP1 R agonists decrease eosinophilia and ILC2 among other mechanisms.
Pite et al. [8]	Review Article	Obesity is a risk factor for asthma that is associated with more refractory disease. Hyperinsulinemia is also related to asthma.
Khan et al. [9]	Human Model	GLP1 receptor agonists led to weight loss and decreased asthma exacerbations.
Vara et al. [10]	Human Model	GLP1 receptor agonists increase surfactant production in type II pneumocytes.
Romaní-Pérez et al. [11]	Animal Model	GLP1 receptor agonist given to pregnant rats increased surfactant production in rats after birth.
Foer et al. [12]	Human Model	Periostin levels were found to be lowered in asthmatics diabetics on GLP-1RAs
Suzuki et al. [13]	Animal Model	Vildagliptin ameliorates pulmonary fibrosis in lipopolysaccharide-induced lung injury by inhibiting endothelial-to-mesenchymal transition
Foer et al. [14]	Retrospective cohort study	Patients with asthma prescribed GLP1 R agonists for diabetes had fewer asthma exacerbations.
Forno [15]	Commentary	Commentary to Foer et al., suggesting further research be done comparing asthma and obesity patients on GLP-1 RAs and metformin. Further data needed to define asthma exacerbations.
Toki et al. [16]	Animal Model	GLP1 Receptor Agonists may be a novel therapy for asthma by reducing aeroallergen-induced neutrophilia. GLP-1 RAs decrease lung protein excretion of IL 5 and 13.
Hur et al. [17]	Animal Model	GLP1 receptor agonist decreased interleukin 4, 5 and 33 in bronchial fluid. GLP1 receptor agonist suppressed peri bronchial inflammation. Obese asthmatic mice have increased expression of NLRP3, activated Caspase 1 and IL 1B that was reduced by GLP1 receptor agonists.
Fandiño et al. [18]	Animal Model	GLP1 Receptor agonists increased surfactant production and partially restored lung alveolar function in pulmonary fibrosis.
Mitchell et al. [19]	Animal Model	GLP1 agonists were found to decrease eosinophilic secretion of interleukin 4, interleukin 8, and interleukin 13 in mice with asthma.

Evidence supporting the anti-inflammatory effects of GLP-1-RAs in Asthma

**Figure 1:**
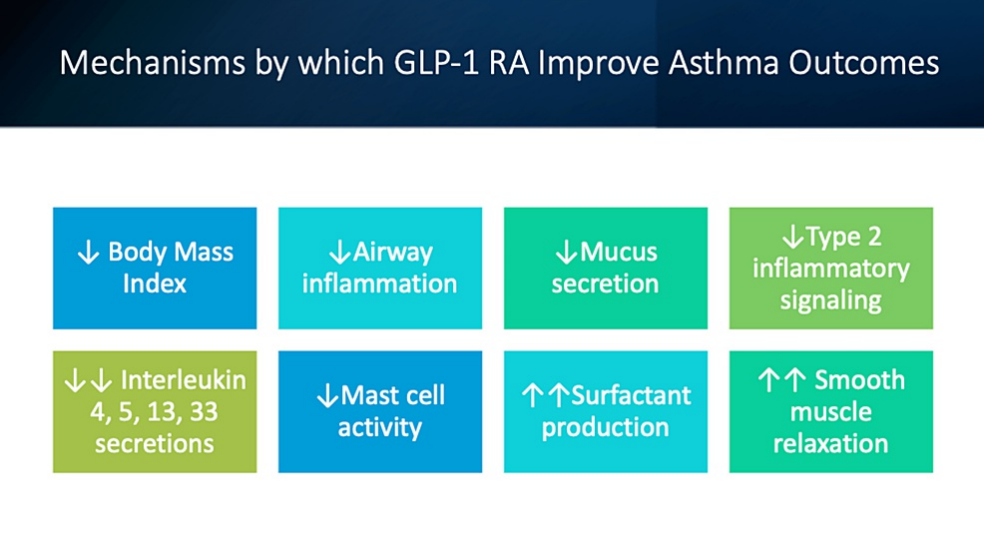
Mechanisms by which GLP-1 RAs Improve Asthma Outcomes GLP-1 RAs: Glucagon-like peptide-1 receptor agonists

Evidence supporting the anti-inflammatory effects of GLP-1 RAs mostly from basic science studies showed GLP-1 RAs salutary effects on mediators of inflammation and pathological remodeling in asthma. In their study based on an animal model, Oztay et al. showed a significant improvement in pulmonary edema, a reduction in glucose-mediated oxidative stress and moderating effect on hyperglycemic lung damage in diabetic mice exposed to GLP-1 RAs [4]. Toki et al. demonstrated that GLP-1 RAs use was associated with a substantial effect in reducing aeroallergen-induced neutrophilia, and decreasing lung protein excretion of IL 5 and 13 [16]. Similarly, Hur et al. found a reduction in IL 4, 5 and 33 in bronchial fluid after exposure to GLP-1 RAs, along with a reduction in peri-bronchial inflammation, and a reduction in the expression of NLRP3, activated Caspase 1 and IL 1B in obese asthmatic mice [17]. Finally, Mitchell et al. showed a reduction in secretion of IL4, IL8, and IL13 by eosinophils in asthmatic mice, pointing to putative mechanisms for the beneficial effects of GLP-1 RAs in asthma (Figure 1) [19].

Other evidence of beneficial effects of GLP-1 RAs use is derived from increased surfactant production. Fandiño et al. showed an increase in surfactant production and subsequent restoration (albeit partial) of lung alveolar function in pulmonary fibrosis [18]. Vara et al. also found GLP-1 RAs increase surfactant production in type II pneumocytes, a finding similar to a study by Romaní-Pérez et al., which showed that pregnant rats had a rise in surfactant production-post-delivery [10-11]. The evidence from basic science is in support of the positive findings by Foer et al., in their retrospective cohort study of patients with asthma who are on GLP-1 RAs. In this study, GLP-1 RAs use was associated with fewer exacerbations of asthma [14].

## Conclusions

The evidence we presented attests to the basis of our recommendation of early use of GLP-1 RAs in asthma. The evidence and data from animal, basic science, epidemiological and clinical studies highlight the pleiotropic properties and benefits of GLP-1 RAs in reducing airway inflammation, pathological remodeling, and other pathobiological mechanisms underpinning asthma - beyond the well-known GLP-1 RA roles in treating diabetes and obesity. Preclinical studies are suggesting a potential role of GLP-1 RAs in reducing pulmonary inflammatory response via signaling pathways.

Reduction in asthma exacerbations and symptoms may be achieved with GLP-1 RAs, irrespective of weight loss and target glucose control. Current literature suggests that GLP-1 agonism may potentially have a systemic anti-inflammatory effect and merit further studies in the treatment of asthma. GLP-1 RAs are affordable, minimal-risk medications compared to the current methods of asthma treatment. The pathophysiology of patients with asthma and diabetes may be different than those with asthma from childhood, and randomized clinical trials are ongoing. Further understanding of GLP-1 receptor signaling in the lung and how it relates to asthma and metabolic syndrome will help guide targeted therapies for asthmatics. Thus, early use of GLP-1 RAs - in the setting of asthma and diabetes/metabolic syndrome - can potentially lead to better asthma outcomes, less medication burden, lower cost, and overall improvement of asthma-related quality of life measures. There is an urgent need for large randomized, controlled, clinically comparative trials of different GLP-1 RAs in the setting of co-occurring asthma, diabetes, and/or obesity. In sum, we recommend that all clinicians (MDs, NPs, and PA) strongly consider the use of GLP-1 RAs in the early stage of asthma in obese diabetics to prevent or slow down the beginning of pathological remodeling and permanent lung damage.
